# Salmon Consumption Behavior Prediction Based on Bayesian Optimization and Explainable Artificial Intelligence

**DOI:** 10.3390/foods14030429

**Published:** 2025-01-28

**Authors:** Zhan Wu, Sina Cha, Chunxiao Wang, Tinghong Qu, Zongfeng Zou

**Affiliations:** 1 School of Economics and Management, Shanghai Ocean University, Shanghai 201306, China; m220751363@st.shou.edu.cn; 2 School of Business, The University of Hong Kong, Hong Kong 999077, China; chasina@connect.hku.hk; 3 School of Management, Shanghai University, Shanghai 200444, China; zfzou@mail.shu.edu.cn

**Keywords:** machine learning, SHAP model, consumption behavior prediction, influencing factors, salmon

## Abstract

Predicting seafood consumption behavior is essential for fishing companies to adjust their production plans and marketing strategies. To achieve accurate predictions, this paper introduces a model for forecasting seafood consumption behavior based on an interpretable machine learning algorithm. Additionally, the Shapley Additive exPlanation (SHAP) model and the Accumulated Local Effects (ALE) plot were integrated to provide a detailed analysis of the factors influencing Shanghai residents’ intentions to purchase salmon. In this study, we constructed nine regression prediction models, including ANN, Decision Tree, GBDT, Random Forest, AdaBoost, XGBoost, LightGBM, CatBoost, and NGBoost, to predict the consumers’ intentions to purchase salmon and to compare their predictive performance. In addition, Bayesian optimization algorithm is used to optimize the hyperparameters of the optimal regression prediction model to improve the model prediction accuracy. Finally, the SHAP model was used to analyze the key factors and interactions affecting the consumers’ willingness to purchase salmon, and the Accumulated Local Effects plot was used to show the specific prediction patterns of different influences on salmon consumption. The results of the study show that salmon farming safety and ease of cooking have significant nonlinear effects on salmon consumption; the BO-CatBoost nonlinear regression prediction model demonstrates superior performance compared to the benchmark model, with the test set exhibiting RMSE, MSE, MAE, R^2^ and TIC values of 0.155, 0.024, 0.097, 0.902, and 0.313, respectively. This study can provide technical support for suppliers in the salmon value chain and help their decision-making to adjust their corporate production plan and marketing activities

## 1. Introduction

China is the largest consumer of aquatic products in the world, and the demand for high-quality aquatic products has increased drastically with the rising disposable income of Chinese urban residents and their increasing concern for dietary health issues [1,2]. Among the many aquatic products, salmon is increasingly popular among consumers due to its taste and rich nutritional value, especially its high-protein, low-fat properties and richness in Omega-3 fatty acids. With consumers’ continuous pursuit of health and quality, the consumption trend of salmon in China will be further expanded, and the market potential is huge [3,4]. The study of factors affecting the consumers’ purchasing behavior and its prediction are not only conducive to improving the consumption level of salmon consumers in China, but also to optimizing the dietary structure of consumers. At the same time, it is beneficial for seafood suppliers to adjust their production and marketing plans according to consumers’ preferences in order to increase their profits and market shares, which are of great significance in promoting the high-quality development of China’s fishing industry.

In recent years, scholars at home and abroad, based on the theory of planned behavior and the theory of consumer behavior, have mainly used the structural equation model, the logistic regression (Logistic) model, and the multivariate probability ratio regression (Probit) model [5,6,7,8] to study the consumers’ behavior when purchasing aquatic products and the factors influencing it from different perspectives. Meng et al. used the Tobit model to study the key factors affecting aquatic product consumption among Shanghai residents, and the study showed that income, aquatic product safety perception, and quality preference were important determinants of aquatic product purchase among Shanghai residents [9]. Fish consumption preference is negatively correlated with family size and positively correlated with age, education level, and income level [10,11,12]. In terms of purchasing channels, consumers have different types of behavioral preferences when purchasing at farmers’ markets, supermarkets, etc., Skallerud et al. explored the consumers’ store choice behavior for purchasing Norwegian salmon in a cross-cultural context, and found that the consumers’ culture, education, family size, employment status, product preferences, and attitudes toward the product were highly correlated with their choices of different retail outlets when purchasing Norwegian salmon [13]. Furthermore, in terms of salmon safety, the proportion of salmon purchased with certification and traceability is increasing as the consumers’ awareness continues to grow. Haghiri et al. showed that the likelihood of paying a high price for a certified product increases when food quality attributes and sustainable agricultural practices, are combined with the concept of food safety [14]. Britwum et al. used the Multinomial logit model to assess the U.S. consumers’ willingness to pay for farmed salmon and found that certified farmed seafood has a higher price premium, as the people’s confidence in the safety of farmed seafood increases [15]. Zhang et al. used the ordered probit model and the two-limit Tobit model to study the seafood consumption patterns of the residents of six provinces and cities in China, and found that health motivation is an important predictor of seafood consumption [16].

However, consumption behavior constitutes a multifaceted, coupled, nonlinear system, rendering traditional statistical methods inadequate for capturing its intricate nonlinear relationships and accurately forecasting consumer actions [17,18,19]. Conversely, machine learning models exhibit robust nonlinear fitting and adaptive capabilities, efficiently mining complex features within multidimensional and redundant datasets, thereby finding application in the domain of consumer behavior analysis [20,21]. In recent years, there has been a surge in research leveraging machine learning to predict consumer preferences and future purchasing patterns. Kumar and colleagues developed a predictive model for consumer repurchase intentions, utilizing the Artificial Bee Colony (ABC) algorithm, alongside five machine learning algorithms. Their findings revealed that the AdaBoost model surpassed neural networks and supported the vector machines’ performance [22]. Trivedi and team formulated an interpretable improved stacked SVM (XISS) model to forecast consumer refill frequencies for liquefied petroleum gas (LPG), achieving a prediction accuracy of up to 81% [23]. Andoh-Odoom et al. employed classification and regression trees to anticipate factors influencing consumer preferences, purchasing behaviors, and willingness to purchase tilapia fillets, validating the model’s efficacy [24].

Compared with traditional machine learning models, Extreme Gradient Boosting (XGBoost) is an integrated gradient boosting learning algorithm, which can reveal the underlying mechanism between input features and target results, and can effectively deal with abnormal, non-linear, high-dimensional data, with advantages such as a high prediction accuracy and less overfitting [25]. Hu W. et al. showed that the Random Forest algorithm is more conducive to dealing with complex nonlinear systems that objectively reflects the indicator’s contribution [26]. Lei, Y. et al. applied the XGBoost model to quantify the importance of each variable for the monthly fire risk level [27]. In addition, as interpretable machine learning becomes more popular, machine learning methods can extract unique insights from large datasets with a large number of feature variables [28,29]. Wang M. et al. used Shenzhen City as a research object, constructed an XGBoost model, combined it with Shapley additive explanatory maps and partial dependency maps, and showed that the mean building volume was a key parameter, with an average SHAP value of 0.0107 m and a contribution rate of 9.70% [30].

In conclusion, the visual and quantitative capabilities of machine learning that can be explained can be used to further study the driving mechanism that affects consumers’ purchase intention. However, the current domestic and foreign literature on the factors affecting the consumption of aquatic products mainly focuses on the willingness to pay and on purchase behavior of aquatic products. Most studies focus on the broad categories of aquatic products. Few people study the purchase factors of specific seafood, and few studies use interpretable machine learning to predict and analyze the driving factors of seafood consumption behavior. Therefore, using the research on the salmon consumption behavior of Shanghai residents, a prediction model of seafood consumption behavior based on nine algorithms including neural network, XGBoost, LightGBM, CatBoost, and NGBoost, was established. SHAP was used to assess the effect sizes and interaction effects of different factors, and the Accumulated Local Effects Plot was used to analyze the specific prediction patterns of the characteristic variables, such as aquaculture safety and the individual’s household income, on salmon consumption. The possible marginal contributions of this paper lie in comprehensively applying multiple machine learning methods to study the salmon consumption problem, thus enriching the related research; more accurately revealing the complex relationships among variables when exploring the influencing factors of salmon consumption by employing cutting edge Ensemble learning; and utilizing the interpretable methods in machine learning, exploring the significance of different influencing factors on salmon consumption, and analyze the specific prediction models subject to salmon quality attributes and other important influencing factors, which has important reference value and technical support for promoting the high-quality development of salmon enterprises and for the suppliers in the salmon value chain to adjust their enterprise production plans and marketing activities.

## 2. Materials and Methods

### 2.1. Bayesian Optimization Algorithm

The Bayesian optimization (BO) algorithm, as a versatile and powerful tool, has demonstrated its outstanding application value in many fields, such as optimizing robot mechanics, environmental monitoring, and material design [31,32,33]. Furthermore, in terms of hyperparametric optimization for machine learning algorithms, the work of Feurer and Hutter in 2019 further confirmed the effectiveness of Bayesian optimization algorithms [34].

Bayesian optimization is a global optimization algorithm proposed by Pelikan et al. [35] to find the optimal solution to minimize the objective function in a high-dimensional non-convex search space, where the posterior distribution of the objective function (Gaussian process) is updated by continuously adding sample points without knowing the internal structure and mathematical properties of the optimized objective function until the posterior distribution fits the real distribution. The objective function of the Bayesian optimization algorithm is as follows:

(1) $$X^{\left(*\right)}=arg\;min{\;X}_{x\in s}\;f(X)$$

where $X^{\left(*\right)}$ denotes the optimal set of hyperparameters; *S* represents the candidate set of *x*; and f(X) is the objective function.

### 2.2. Boosting Regression Algorithm

The Extreme Gradient Boosting (XGBoost) algorithm is an ensemble learning algorithm that has been widely used in the field of machine learning in recent years, and was developed by Tianqi Chen et al. [36]. It is based on the idea of Gradient Boosting Decision Tree (GBDT), and constructs a stronger supervised model by combining multiple weak learners to reduce the learning error and updating the sample weights during each iteration.

The core strength of the XGBoost algorithm lies in the second-order Taylor expansion of the loss function and the introduction of a regularization term, which work together to prevent the overfitting of the model and thus improve its generalization ability [37]. The mathematical principle of the XGBoost algorithm can be summarized as follows:

(2) $$\hat{y_{i}}=\sum_{k=1}^{k}f_{k}\left(x_{i}\right),\;f_{k}\in F\;$$

The XGBoost constructs the model by iteratively adding CARTs (Classification and Regression Trees), where *k* is the number of CARTs and *f_k_* is a function in the function space *F* in Equation (2). At each iteration, XGBoost adds a new tree model to reduce the residuals. The optimization objective function required for the XGBoost regression model is as follows.

(3) $${\mathrm{obj}}^{\left(t\right)}=L\left(y_{i}\;,\;{\hat{y_{i}}}^{t}\;\right)+\Omega \;\left(f_{t}\right)$$

where, $L\left(y_{i},\;{\hat{y_{i}}}^{t}\;\right)$ is the training loss function, which is used to measure the predictive ability of the model. $\Omega \left(f_{t}\right)$ is the regularization term, related to the complexity of the tree, used to control the complexity of the model to prevent overfitting. The formula is as follows:

(4) $$\Omega \left(f\right)=\gamma T+\frac{1}{2}\lambda \sum_{j=1}^{T}w_{j}^{2\;}$$

where *T* is the number of leaf nodes, *w_j_* is the score of the jth leaf node, and *γ* and *λ* are regularization parameters controlling the number of leaf nodes and the size of leaf node weights, respectively. In this paper, we use the mean square error (MSE) as the loss function and the objective becomes as follows

(5) $$\begin{array}{rr} {obj}^{\left(t\right)} & =\sum_{i=1}^{n}{\left(y_{i}-\left({\hat{y}}_{i}^{\left(t-1\right)}+f_{t}\left(x_{i}\right)\right)\right)}^{2}+\sum_{i=1}^{t}\omega \left(f_{i}\right)\\ & =\sum_{i=1}^{n}\left[2\left({\hat{y}}_{i}^{\left(t-1\right)}-y_{i}\right)f_{t}\left(x_{i}\right)+f_{t}{\left(x_{i}\right)}^{2}\right]+\omega \left(f_{t}\right)+constant \end{array}$$

To facilitate the solution, we perform a second-order Taylor expansion of the loss function of the XGBoost model to obtain an approximation of the objective function in quadratic form.

(6) $${obj}^{\left(t\right)}=\sum_{i=1}^{n}\left[l\left(y_{i},{\hat{y}}_{i}^{\left(t-1\right)}\right)+g_{i}f_{t}\left(x_{i}\right)+\frac{1}{2}h_{i}f_{t}^{2}\left(x_{i}\right)\right]+\omega \left(f_{t}\right)+constant$$

(7) $$g_{i}=\partial_{{\hat{y}}_{i}^{\left(t-1\right)}}l\left(y_{i},{\hat{y}}_{i}^{\left(t-1\right)}\right)$$

(8) $$h_{i}=\partial_{{\hat{y}}_{i}^{\left(t-1\right)}}^{2}l\left(y_{i},{\hat{y}}_{i}^{\left(t-1\right)}\right)$$

where *g_i_* and *h_i_* are the first-order derivative (gradient) and second-order derivative (Hessian matrix) of the loss function with respect to the predicted value, respectively. In XGBoost, gi and hi denote the gradient and curvature of the current prediction value, respectively.

XGBoost uses greedy algorithms to build the tree models step by step. In each iteration step a new tree model is learned to reduce the objective function. The model updates weights through leaf split and leaf weight. At each dividing point, the gain of the dividing point is calculated. Gain indicates the reduction in error after segmentation. For the split point, the gain can be expressed as:

(9) $$\mathrm{Gain}=\frac{1}{2}\left[\frac{G_{L}^{2}}{H_{L}+\lambda }+\frac{G_{R}^{2}}{H_{R}+\lambda }-\frac{{\left(G_{L}+G_{R}\right)}^{2}}{H_{L}+H_{R}+\lambda }\right]-\gamma$$

where $G_{L}$ and $G_{R}$ are the sets of samples from the left and right subtrees after treeclassification, $G_{L}^{2}/\left(H_{L}+\lambda \right)$ is the information score of the left subtree, $G_{R}^{2}/\left(H_{R}+\lambda \right)$ is the information score of the right subtree, and ${\left(G_{L}+G_{R}\right)}^{2}/\left(H_{L}+H_{R}+\lambda \right)$ is the information score of the current unsegmented tree.

The LightGBM algorithm, developed by Microsoft, employs a histogram-based decision tree approach to enhance computational efficiency [38]. This method involves discretizing continuous feature values into a finite number of bins, thereby simplifying the process of identifying optimal splits. By focusing on the leaf-wise growth strategy, LightGBM iteratively selects the leaf node with the highest gain for splitting, which not only accelerates tree growth but also improves model training efficiency. This strategy also contributes to superior data fitting, leading to enhanced model accuracy.

CatBoost, developed by Yandex, is a gradient boosting framework that employs oblivious decision trees as its fundamental learner [39]. The CatBoost algorithm combines classification and boosting techniques to efficiently process features according to category. It solves problems related to gradient and prediction bias. CatBoost can automatically process categorical features without converting them to numerical features.

It processes the ordering information of the categorical features through a technique called “ordered boosting” and uses this information in the tree splitting process.In addition, CatBoost features an adaptive learning rate, which can adaptively adjust the learning rate according to different features. The adaptive learning rate is calculated as follows:

(10) $$\eta_{t}=\frac{1}{t+\lambda }$$

(11) $$\alpha_{t}=\frac{\sum_{i=1}^{t}\eta_{i}}{t}$$

where *t* is the number of iterations, *η_t_* is the learning rate of the tth round of iterations, *α_t_* is the average learning rate of the previous *t* rounds of iterations, and *λ* is a regularization parameter. The adaptive learning rate helps the algorithm to better control the contribution of weak learners in each round of iterations, thus improving the accuracy of the whole model.

### 2.3. SHapley Additive ExPlanations Model and the Accumulated Local Effects Plot

The SHAP (SHapley Additive exPlanations) model is a method for interpreting the predictions of machine learning models, proposed by Lundberg and Lee in 2017 [40]. The SHAP values are based on the Shapley values from game theory, and are used to equitably distribute each participant’s contribution to the collective outcome. The SHAP values have several key properties, including efficiency, symmetry, virtuality, and additivity [41]. These properties ensure that the sum of the characteristic contributions is equal to the difference between the predicted value and the mean, and that the aggregation of predictions from a single model is equal to the predictions of all the models combined. The formula for calculating the SHAP value is shown below:

(12) $$y_{i}=y_{\mathrm{base}}+f\left(x_{i1}\right)+f\left(x_{i2}\right)+\cdots +f\left(x_{\mathrm{ij}}\right)$$

where *y_base_* is the average value of the target variable over all samples; *f*(*x_ij_*) is the SHAP value of *x_ij_*. The calculation of SHAP values involves multiplying the marginal contribution of each feature by the corresponding weight and then summing. This method not only reflects the contribution of features in each sample, but also shows the positivity and negativity of the effect. In this study, the SHAP algorithm, which is based on game theory, was used to interpret and analyze the model. The algorithm possesses consistency and local accuracy and can effectively interpret the results of machine learning prediction models. This approach can provide deeper understanding and insight into consumer behavior prediction research, which can help optimize the model prediction results and improve user trust and satisfaction.

The LIME method may be influenced by randomness and sampling techniques when interpreting model predictions, potentially resulting in unstable interpretation outcomes. While the CAM method offers the model’s attentional weights for various spatial locations, its interpretive scope is relatively confined to a specific deep learning model. Consequently, the SHAP method holds a relative edge over LIME and CAM in terms of interpretability, stability, and scope [42].

In this paper, ALE plots are used to describe the specific prediction patterns of key influencing factors on salmon. ALE plots eliminate the influence of variable correlation by calculating local effects. First, the range of values of the characteristic variables is divided into intervals, ensuring that each interval has the same number of data points. Second, the local effects within each interval are calculated, which results in the value of the cumulative local effects of the feature variables on the model predictions [43,44].

### 2.4. Model Construction and Experimental Process

In this study, the original data were randomly divided into 319 sets of training set samples and 80 sets of test set samples using python’s scikit-learn, and were used as input features for nine regression prediction models, including XGBoost, LitghtGBM, CatBoost, and NGBoost, based on the consumers’ personal characteristics and aquatic product attributes to predict salmon consumption behavior Finally, the SHAP model and the ALE plot were used to analyze the regression prediction results of the optimal model and to explore the key factors influencing consumers to purchase salmon. The experimental environment of the study was Python 3.8, TensorFlow 1.15.5, and Pytorch 1.11.0. To ensure the consistency of the data scale, the data were normalized and uniformly mapped into the interval of [0,1]. The normalization formula is as follows:

(13) $$X=\frac{X_{i}-X_{min}}{X_{max}-X_{min}}$$

where *X* and *X_i_* are the data after and before normalization, respectively.

In addition, a series of important third-party libraries are introduced. Among them, the Sklearn machine learning library provides rich machine learning algorithms and tools for data processing and model evaluation; the SHAP library provides two modules for global analysis and local analysis. To more comprehensively and accurately evaluate the effectiveness of the proposed model, several widely recognized statistical metrics were utilized, including mean absolute error (MAE), mean square error (MSE), root mean square error (RMSE), R-squared (R^2^) and Terrell’s Inequality Coefficient (TIC). These indicators are commonly employed to assess the predictive performance of models, with smaller values signifying lower prediction errors and, consequently, higher prediction accuracy. The mathematical expressions for these metrics are presented as follows:

(14) $$\mathrm{MSE}=\frac{1}{n}\;\sum_{i=1}^{n}{\left(y_{i}-{\hat{y}}_{i}\right)}^{2}$$

(15) $$\mathrm{MAE}=\frac{1}{n}\sum_{i=1}^{n}\left|y_{i}-{\;\hat{y}}_{i}\right|$$

(16) $$\mathrm{RMSE}=\sqrt{\frac{1}{n}\sum_{i=1}^{n}{\left(y_{i}-\;{\hat{y}}_{i}\right)}^{2}}$$

(17) $$R^{2}=1-\frac{\sum_{i=1}^{n}{\left(y_{i}-{\hat{y}}_{i}\right)}^{2}}{\sum_{i=1}^{n}{\left(y_{i}-\overset{-}{y}\right)}^{2}}$$

(18) $$\mathrm{TIC}=\frac{\sqrt{\frac{1}{n}\sum_{i=1}^{n}{\left(y_{i}-{\hat{y}}_{i}\right)}^{2}}}{\sqrt{\frac{1}{n}\sum_{i=1}^{n}y_{i}^{2}}+\sqrt{\frac{1}{n}\sum_{i=1}^{n}{{\hat{y}}_{i}}^{2}}}$$

where $y_{i}$ and ${\hat{y}}_{i}$ are the actual and predicted values at time *i*, respectively; *n* is the number of predicted data.

## 3. Data Sources and Analysis

### 3.1. Data Sources

This study takes Shanghai residents as the research object, combines the related literature [45,46,47], and designs the questionnaire. The research questionnaire includes three main parts. The first part includes individual consumer characteristics, such as gender, age, education level, household income, and family demographics. The second part includes the consumers’ perception of salmon, including four aspects of the commodity: nutritional quality, safety quality, sensory quality, and added value. The third part deals with the consumers’ willingness to consume salmon. The formal questionnaire survey was initiated after the questionnaire was thoroughly revised and free of errors. The research data were obtained through a combination of online and offline methods: online through the Questionnaire Star web-based program; offline through the random distribution of questionnaires to passers-by in residential areas and major supermarkets, filling out the questionnaires on the spot, and then recycling them to carry out the research. A total of 450 questionnaires were distributed, of which 399 were valid, accounting for 88.67%. In addition, regarding the sample size, we followed the rule of thumb for machine learning models that the sample size should be at least 10 to 100 times the number of attributes/features considered in the study [19,48]. A total of 20 feature variables were considered in this study to predict salmon purchase intention. Therefore, 399 samples met the a priori condition.

### 3.2. Basic Consumer Characteristics

The basic information of the respondents is shown in Table 1. In terms of gender, males and females accounted for 40.9% and 59.1% of the total samples, respectively, indicating that females are the majority of the buyers of salmon. Next, the age group of 21 to 44 years accounted for 77.65% of the total sample. The average education level of the respondents was relatively high, with the majority holding a bachelor’s degree or higher. Monthly household income was mainly between $5000 and $10,000, and the monthly household income was mainly between $10,001 and $20,000, accounting for 7% and 9.5% of the entire sample, respectively.

## 4. Results

### 4.1. Variable Description and Assignment

This paper selects whether consumers buy salmon as an explained variable and assigned values of 0 and 1. The naming and assignment of other independent variables are shown in Table 2. In order to reproduce the experiment in this paper, the number of random seeds in the dataset partition is 291. Nine regression prediction models, such as ANN, XGBoost, LightGBM, CatBoost, and NGBoost, are set as the default parameters of the Skelearn machine learning library. The number of random seeds is set to 100. The prediction performance accuracy of the nine models is compared in the test set, and the optimal prediction model is selected.

### 4.2. Regression Prediction Model Performance Analysis

In addition to the random seed setting, we also paid attention to the consistency of other key parameters, including the selection of evaluation metrics, the size of the training batch, and the setting of the training period. The uniform configuration of these parameters provides a standardized environment for our model training and evaluation, making comparisons between different models fairer and more accurate. Under these strict experimental control conditions, we obtained the prediction performance results of the benchmark model, and these results are shown in Table 3.

RMSE, MSE, MAE, R^2^ and TIC are key metrics to evaluate the performance of a model. The lower the RMSE value, the lower the prediction error; the closer the R^2^ value is to 1, the better the model’s explanatory ability. This study compares nine machine learning models, including ANN, XGBoost, LightGBM and CatBoost. The results show that the R^2^ value of AdaBoost model is 0.734, which is the lowest among the four models, and the TIC value is 0.515 with the largest error. The RMSE, MSE, MAE, R^2^ and TIC values are 0.877 for the CatBoost regression prediction model. The RMSE, MSE, MAE, R^2^ and TIC of the CatBoost regression prediction model were 0.164, 0.027, 0.093, 0.889 and 0.332, respectively, which indicated that its prediction error was relatively small, and the model prediction accuracy was better than that of the other benchmark models. Given the superior performance of the CatBoost regression prediction algorithm in the prediction of salmon consumption behavior, we chose the CatBoost model and combined it with the SHAP (SHapley Additive exPlanations) model to conduct an in-depth analysis of the key factors affecting salmon purchase behavior.

**Figure 1:**
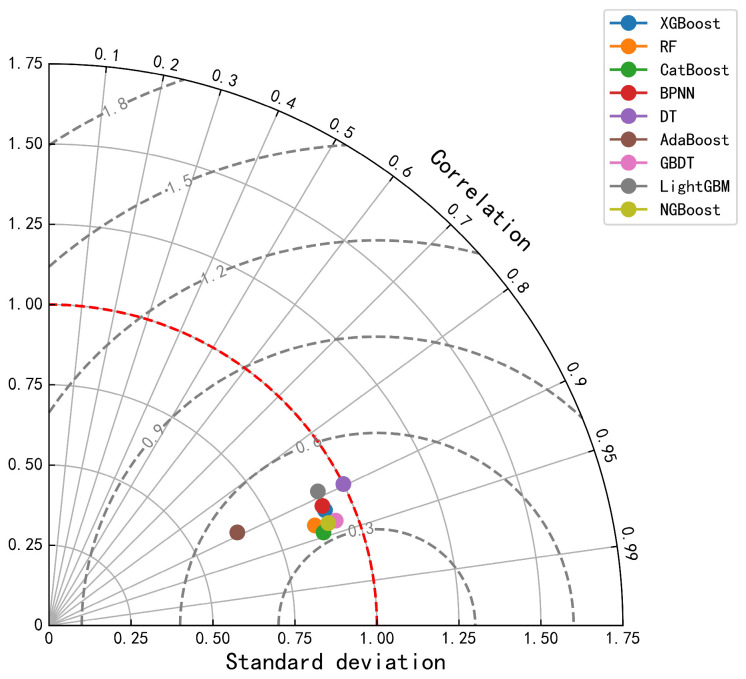
Taylor diagrams for regression prediction models. The gray dashed line is the RMSE contour. The red dashed line is the reference line with a standard deviation of 1, which is used to quickly determine prediction accuracy.

Figure 1 provides a comprehensive assessment of the nine regression prediction models’ performance using Taylor plots. Taylor plots summarize multiple aspects of model performance in a single plot, including correlation coefficients, normalized standard deviations, and distances from the observations [49,50]. The gray dashed line is the RMSE contour. The red dashed line is the reference line with a standard deviation of 1, which is used to quickly determine prediction accuracy. According to Figure 1, the CatBoost model provides the best prediction performance.

To avoid the overfitting problem of the CatBoost model, the model parameters must be optimized. In CatBoost hyperparameter tuning, the Bayesian optimization method automatically finds the best hyperparameter configuration to minimize the model validation error [51,52]. In this paper, we identify the most important parameters and value ranges that may affect the validity of the CatBoost model. These parameters and value ranges will be replaced in the Bayesian optimization algorithm. The combinations of the hyperparameters used for the CatBoost model prediction are as follows: the optimal range of depth is [3, 10]; the optimal range of depth is [1, 500]; and the optimal range of learning rate is [0.01, 0.3]. The specific parameters are shown in Table 4.

As shown in Table 5, the RMSE, MSE, MAE, R^2^, and TIC of the BO-CatBoost prediction set are 0.155, 0.024, 0.097, 0.902, and 0.313, respectively, which are better than that of the benchmark CatBoost prediction model. The R^2^ and TIC of the BO-CatBoost model are 1.46 percent better than that of the benchmark CatBoost prediction model. The R^2^ and TIC of the BO-CatBoost model are 1.46% higher and 5.72% lower than those of the benchmark CatBoost model, respectively.

**Figure 2:**
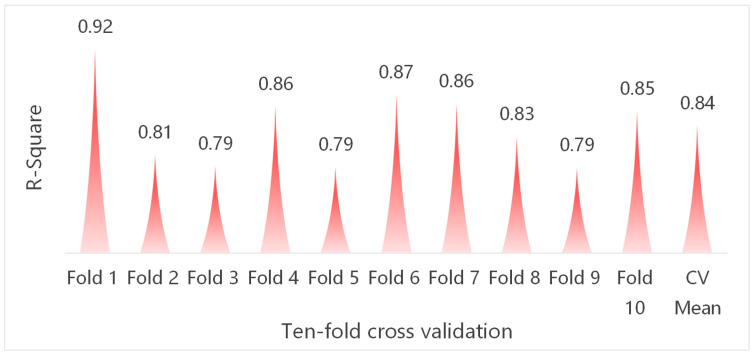
Cross-validation results.

In this paper, the tenfold cross-validation method is used to test whether the model has the overfitting phenomenon, as shown in Figure 2. The tenfold cross-validation method means that the data are divided into ten parts, nine of which are used as training data, and the remaining part is used as test data, and the cycle is repeated ten times to ensure that each set of data is tested once [53]. The final evaluation result is the average of the ten evaluations. The results of the tenfold cross-validation show that the average value of R^2^ for the test set of the CatBoost model is 0.84, indicating that the CatBoost model has good regression prediction performance and robustness.

### 4.3. SHAP Modeling and ALE Analysis

**Figure 3:**
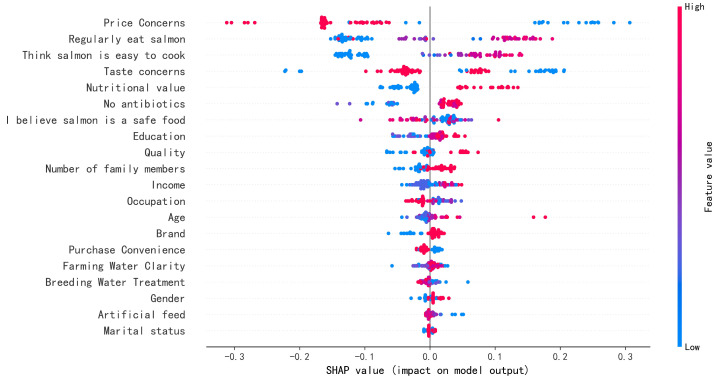
SHAP summary plot.

Figure 3 presents the global feature analysis of the CatBoost model using SHAP values. Key predictors of salmon consumption include consumer habits, price, and the use of antibiotics in farming practices. To gain a more intuitive understanding of how these features influence the model’s output, extract valuable insights, and assist companies in implementing targeted marketing strategies, this study employs a SHAP value mapping diagram to illustrate the nonlinear relationships among variables [48]. Unlike partial dependency plots, the vertical axis of the SHAP value mapping diagram represents the SHAP value itself, rather than the labeled output value. This approach allows for a more effective analysis of the nonlinear impacts of significant predictors on salmon consumption.

From the respondents’ viewpoint on the quality and safety factors in salmon farming, salmon without medication in the farming process is more popular among consumers; as their living standard improved, the consumers began to pay more attention to the salmon’s quality and safety when purchasing the product. In regard to taste, salmon with good taste presents a negative effect on purchase intention. In regard to quality, consumers are more inclined to choose salmon with good quality. As the living standard of the population has improved, the demand for aquatic products has shifted from quantity to quality, and consumers will choose the best quality products within their price range. From the perspective of respondents’ marketing concerns for salmon, a reasonable price has a significant impact on the consumers’ willingness to buy. The consumers’ willingness to purchase is negatively impacted by reasonable pricing. Since salmon is a more expensive aquatic product than other aquatic products, there is a negative correlation between price and consumers’ willingness to purchase; a lower price for salmon could cause consumers to doubt the product’s quality.

Higher literacy levels correlate with increased consumption of salmon, as individuals with greater literacy are more likely to purchase medium to high-end aquatic products. This demographic tends to possess a better understanding of salmon and generally has a greater economic capacity to afford it. From an income perspective, there is a direct relationship between income levels and salmon consumption; as income rises, so does the demand for salmon. This fish is highly sought after in the domestic market, yet the costs associated with salmon farming and logistics are substantial, coupled with a short shelf life. Consequently, these factors contribute to the elevated price of salmon. Interpretive modeling is established through the design of the model, while the SHAP model elucidates the relationships among the variables. The insights derived from the SHAP model and ALE diagrams do not merely replicate previous findings, even though they align with the conclusions drawn from studies utilizing interpretive modeling.

**Figure 4:**
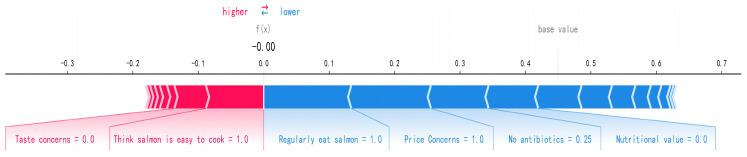
SHAP force plot.

The SHAP method interprets the predictions of an instance by calculating the extent to which each feature in the instance contributes to the prediction. This instance-specific calculation allows SHAP to analyze the predictions of any instance, i.e., to interpret the model locally. Figure 4 shows an explanatory power diagram for a single instance that allows personalized marketing to be assigned to consumers. SHAP labels the degree of contribution of each feature, with blue indicating a negative contribution to salmon consumption and red indicating a positive contribution to salmon consumption [54]. In this instance, taste and ease of cooking provide a positive gain for consumers purchasing salmon; affordable price and safe farming provide negative gains.

**Figure 5:**
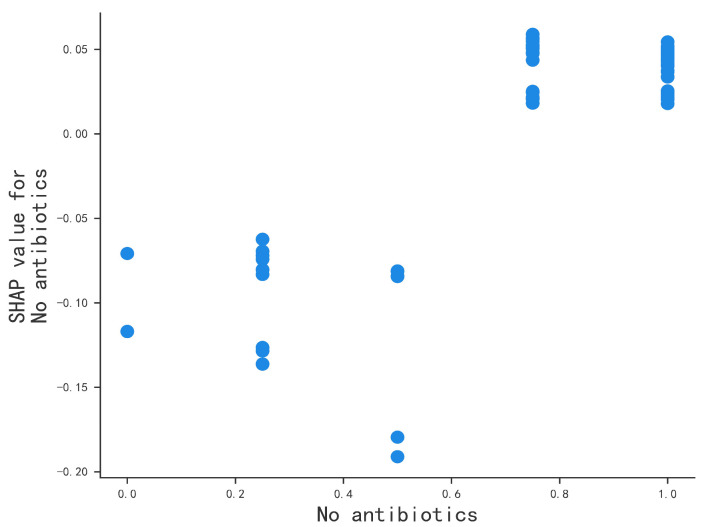
Analysis of SHAP feature dependency plot.

**Figure 6:**
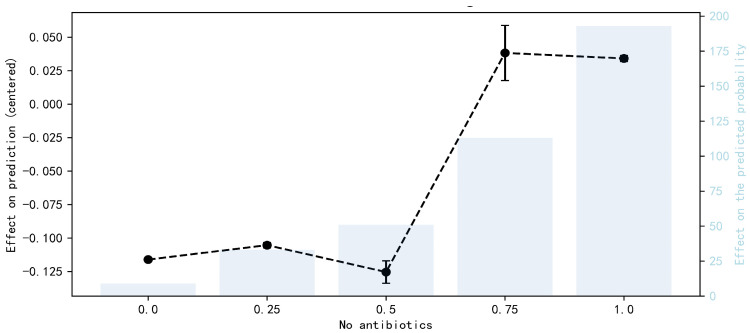
Analysis of accumulated local effects plot.

The one-sample explanation helps to customize the marketing of salmon consumption, and in order to explore the impact of these features on the model output more intuitively, this paper uses SHAP value mapping plots to demonstrate the nonlinear relationship between the variables. Unlike the partial dependency plot, the vertical coordinate of the SHAP value mapping plot is the SHAP value rather than the output label value [55,56]. It can reflect the magnitude of the marginal effect between each variable and salmon consumption. As can be seen from Figure 5 and Figure 6, when the consumer attitude is at level 1 to 3, the SHAP value is negative, and the negative effect increases. When the consumer attitude is greater than level 4, it shows a significant positive gain, indicating that salmon without medication in the aquaculture process is more popular among consumers.

**Figure 7:**
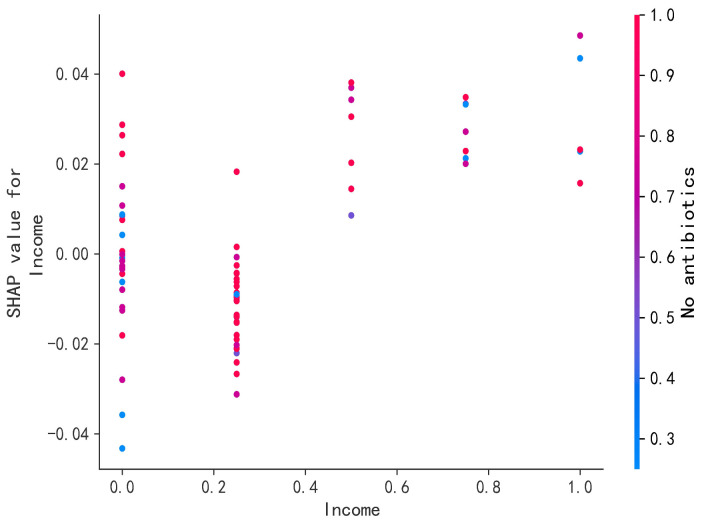
SHAP decision diagram.

This paper analyzes the effects of the consumer income and the farming safety on the willingness to pay for salmon based on the SHAP model, as shown in Figure 7. When consumer income is greater than 10,000 and the external quality attributes of drug-free farmed salmon reach level 3 and above, there is a positive gain in consumer willingness to pay for salmon.

## 5. Conclusions

The fishing industry is one of the most important industries in China, and its development is of great significance in promoting sustainable economic and social development in China and guaranteeing its food security. In this study, an interpretable seafood consumption behavior prediction model based on machine learning is proposed to predict salmon consumption behavior. The results show that the CatBoost model with Bayesian optimization has a high accuracy in seafood consumption behavior prediction and that the RMSE, MSE, MAE, R^2^ and TIC of the test set are 0.155, 0.024, 0.097, 0.902, and 0.313, respectively. The intuitive analysis of the results of the SHAP interpretable instrument shows that gender and education level have less influence on salmon consumption; reasonable price and whether antibiotics are used in the farming process are the key factors influencing the consumption of salmon; the interaction analysis plot of the SHAP model shows that when the consumers’ income is more than 10,000, and there were no antibiotics used in the farming process of salmon, the consumers’ willingness to pay for salmon increases positively.

Given that seafood consumption behavior is influenced by a combination of many factors, it is a great challenge to achieve an accurate prediction of it. In order to improve the accuracy of prediction, future research should focus on the deep integration of machine learning methods with causal inference frameworks. Specifically, on the one hand, the intrinsic correlation between variables should be explored via predictive modeling; on the other hand, the causal mechanism should be constructed and verified via explanatory modeling to achieve the full potential of the complementary advantages of the two in terms of objectives and functions, so as to further deepen and broaden the understanding of the factors influencing salmon consumption.

In addition, multimodal techniques and unsupervised machine learning methods should be combined to explore seafood consumption patterns. Incorporating more types and sources of seafood consumption sample data and focusing on the application of spatial–temporal deep learning and interpretable frameworks in seafood consumption behavior prediction, as well as providing more accurate and in-depth insights for the prediction of seafood consumption behavior should be the aim of future research. 

## Figures and Tables

**Table 1:** Statistical characteristics of respondents.

Demographic Variables	Items	Frequency	Percentage %
Gender	Male	163	40.9
	Female	236	59.1
Age	21–25	88	21.5
	25–34	146	36.6
	35–44	78	19.55
	45–54	67	16.8
	Over 55	20	5
Education	Lower secondary school and below	45	11.3
	Higher secondary school	79	19.8
	Bachelor	176	44.1
	Master	89	22.3
	Phd	10	2.5
Monthly household income	Less than 5000 RMB	133	33.33
	5000–10,000 RMB	161	40.35
	10,000–15,000 RMB	28	7
	15,000–20,000 RMB	38	9.5
	Above 20,000 RMB	39	9.8

**Table 2:** Salmon quality attribute variable definitions and assignments.

Features	Variable	Variable Assignment
Quality and safety attributes	Frequent Salmon Eater	1 = Strongly Disagree, 2 = Disagree, 3 = Comparatively, Disagree 4 = Average, 5 = Agree, 6 = Comparatively, Agree 7 = Strongly Agree
	Farming water is clear	1 = Strongly Disagree, 2 = Disagree, 3 = Average, 4 = Agree, 5 = Strongly Agree
	No artificial feed added to the feed	1 = Strongly Disagree, 2 = Disagree, 3 = Average, 4 = Agree, 5 = Strongly Agree
	No antibiotics	1 = Strongly Disagree, 2 = Disagree, 3 = Average, 4 = Agree, 5 = Strongly Agree
	High nutritional value	1 = Yes, 0 = No
	High quality	1 = Yes, 0 = No
Marketing Attributes	Reasonable price	1 = Yes, 0 = No
	Brand	1 = Yes, 0 = No
	Purchase Convenience	1 = Yes, 0 = No

**Table 3:** Evaluation of regression prediction model performance.

Model Name	RMSE	MSE	MAE	R^2^	TIC
ANN	0.202	0.041	0.124	0.833	0.409
DT	0.224	0.05	0.05	0.795	0.452
RF	0.181	0.033	0.094	0.866	0.366
AdaBoost	0.255	0.065	0.209	0.734	0.515
GBDT	0.173	0.03	0.109	0.877	0.35
XGBoost	0.195	0.038	0.093	0.845	0.394
LightGBM	0.226	0.051	0.141	0.791	0.457
NGBoost	0.174	0.03	0.11	0.876	0.352
CatBoost	0.164	0.027	0.093	0.889	0.332

**Table 4:** Bayesian parametric optimization.

Model Name	Parameter Name	Parameter Setting
BO-CatBoost	depth	3
	iterations	267
	Learning_rate	0.136

**Table 5:** BO-CatBoost Predicted Performance.

Model Name	RMSE	MSE	MAE	R^2^	TIC
CatBoost	0.164	0.027	0.093	0.889	0.332
BO-CatBoost	0.155	0.024	0.097	0.902	0.313

## Data Availability

The data supporting this study’s findings are available from the corresponding author upon reasonable request.
